# Differences in Germination of ACCase-Resistant Biotypes Containing Isoleucine-1781-Leucine Mutation and Susceptible Biotypes of Wild Oat (*Avena sterilis* ssp. *ludoviciana*)

**DOI:** 10.3390/plants10112350

**Published:** 2021-10-30

**Authors:** Fatemeh Benakashani, Jose L. Gonzalez-Andujar, Elias Soltani

**Affiliations:** 1 Department of Agronomy and Plant Breeding Sciences, College of Aburaihan, University of Tehran, Pakdasht 3391653755, Iran; benakashani@ut.ac.ir (F.B.); elias.soltani@ut.ac.ir (E.S.); 2 Department of Crop Protection, Instituto de Agricultura Sostenible (CSIC), 14004 Córdoba, Spain

**Keywords:** ecological costs, germination models, herbicide resistance, hydrotime, target-site resistance

## Abstract

Herbicide resistance can affect seed germination and the optimal conditions required for seed germination, which in turn may impose a fitness cost in resistant populations. Winter wild oat [*Avena sterilis* L. ssp. *ludoviciana* (Durieu) Gillet and Magne] is a serious weed in cereal fields. In this study, the molecular basis of resistance to an ACCase herbicide, clodinafop-propargyl, in four *A. ludoviciana* biotypes was assessed. Germination differences between susceptible (S) and ACCase-resistant biotypes (WR_1_, WR_2_, WR_3_, WR_4_) and the effect of Isoleucine-1781-Leucine mutation on germination were also investigated through germination models. The results indicated that WR_1_ and WR_4_ were very highly resistant (RI > 214.22) to clodinafop-propargyl-contained Isoleucine to Leucine amino acid substitution. However, Isoleucine-1781-Leucine mutation was not detected in other very highly resistant biotypes. Germination studies indicated that resistant biotypes (in particular WR_1_ and WR_4_) had higher base water potentials than the susceptible one. This shows that resistant biotypes need more soil water to initiate their germination. However, the hydrotime constant for germination was higher in resistant biotypes than in the susceptible one in most cases, showing faster germination in susceptible biotypes. ACCase-resistant biotypes containing the Isoleucine-1781-Leucine mutation had lower seed weight but used more seed reserve to produce seedlings. Hence, integrated management practices such as stale seedbed and implementing it at the right time could be used to take advantage of the differential soil water requirement and relatively late germination characteristics of ACCase-resistant biotypes.

## 1. Introduction

Herbicide application is an effective and low-cost method for weed control throughout the world. Unfortunately, the extensive and widespread use of herbicides has resulted in the evolution of resistance in many weed species [1]. A great number of weed species (152 dicots and 111 monocots) present resistance to different families of herbicides [2]. Herbicide resistance in weeds is one of the most common problems, threatening human and animal food production [3]. 

Different mechanisms have been identified that are involved in the resistance of weed species to herbicides [4,5]. That resistance can evolve from variations in weed metabolism pathways and mutations [6], and many studies have shown that mutations in agroecosystems under herbicide selection may exhibit a competitive ability or adaptation cost relative to the susceptible wild-type, in herbicide untreated conditions [7,8]. For example, a single amino acid substitution (Isoleucine to Leucine) in an enzyme at herbicide site of action (Acetyl-CoA carboxylase; ACCase) could change the kinetics and function of the enzyme and cause herbicide resistance in winter wild oat (*Avena sterilis* ssp. *ludoviciana* (Durieu) Nyman) (hereafter referred to as *A. ludoviciana*) [9,10]. ACCase is the enzyme that catalyzes the first committed step in fatty acid synthesis, the carboxylation of acetyl-CoA to malonyl-CoA [11]. In the ACCase gene sequence, seven amino acid substitutions have been observed at different codon positions (Asn2078, Cys2088, Gly2096, Ile1781 and Ile2041) resulting in different herbicide resistance levels [12]. Among these amino acid substitutions, Ile-1781 (in ACCase) are the ones most abundantly found in plant species [12].

Weed biotypes with higher fitness produce more individuals; thus, the fitness difference between resistant biotypes (R) and susceptible biotypes (S) may be due to the difference in fertility, pollen and seed production, and the ability to compete [13].

Many studies have shown that herbicide resistance can affect seed germination and the range of optimal germination conditions [14,15,16,17,18]. Awareness of the dormancy and germination patterns of resistant weed seeds can also help in weed resistance management. Among environmental factors, temperature and water potential mainly impact seed dormancy and germination [19,20]. Hydrotime (HT) models are commonly used to describe seed germination response to water potential [21,22]. The hydrotime constant (θ_H_) can be calculated by a multiplication between time to specific germination fraction and actual water potential (ψ) minus base water potential (ψ_b_). Typically, different germination fractions have different values of ψ_b(g)_ and the latter follow a normal bell curve in a seed population; the median of ψ_b_ (ψ_b(50)_) is the base water potential and the standard deviation of the ψ_b(g)_ (σ_ψb_) which shows germination uniformity. These three parameters (θ_H_, σ_ψb_, Ψ_b(50)_) may be used to explain germination fitness, but no study has proved such a claim before.

Low water potential has adverse effects on germination and seedling growth [23]. It has been reported that heterotrophic seedling growth is influenced by the weight of mobilized seed reserve (MSR) and the conversion efficiency of mobilized (CEM) seed reserve to seedling [24,25,26]. The weight of MSR can be divided into initial seed dry weight (ISDW) and the fraction of seed reserve (FSR), which is mobilized (i.e., the seed depletion ratio). Some authors use these components to investigate the impacts of water and salinity stress on seedling growth [24,26]. Soltani et al. [24] found that the most sensitive component of seedling growth (as affected by drought and salinity stress) is the weight of MSR. Cheng et al. [25] showed that seedling dry weight and MSR increased, while CEM declined during the seed germination process. Zheng and Ma [27] investigated heterotrophic seedling growth of *Bombax ceiba* as affected by seed aging and indicated that MSR and FSR significantly decreased with an increase in the duration of aging. However, their results showed no significant change in CEM with an increase in aging. There is no information on any changes in components of heterotrophic seedling growth between weeds that are either resistant or susceptible to herbicides.

*A. ludoviciana* is an annual member of the Poaceae family. This plant is a serious weed species in cereal fields around the world, whose geographic expansion is expected under climate change [10,28]. *A. ludoviciana* can severely reduce cereal yield [29]. Moderate winter wild oat densities, in the 20–80 panicles m^−2^ range, decreased barley yields by nearly 10% in experiments conducted in central Spain, with yield losses of up to 50% when densities reached 300 panicles m^−2^ [30].

Control of this weed is mainly based on acetyl-CoA carboxylase inhibitor herbicides. The increased application of ACCase herbicides and high initial frequency (6 × 10^−10^ plants) of resistant biotypes significantly affects resistance evolution [31]. To date, 263 resistant species have been reported worldwide [2]. The first cases of ACCase herbicide-resistant wild oat biotypes were found in Persia in 2006 [32,33]. Although resistance to ACCase inhibitors is numerically investigated, more research on the trait differences between resistant and susceptible biotypes would be necessary for resistant weed management. The objectives of this study were: (1) to evaluate the molecular basis for resistance of *A. ludoviciana* biotypes to clodinafop-propargyl as an ACCase herbicide; (2) to detect differences in germination and seedling growth between susceptible and resistant biotypes to ACCase inhibitor herbicide under different water potentials through germination models; and (3) to investigate the effect of Isoleucine-1781-Leucine mutation on germination.

## 2. Materials and Methods

### 2.1. Seed Source

*A. ludoviciana* seeds were collected from wheat fields of Khuzestan province in the Southwest region of Iran during July 2015 (Table 1). We used four populations that were suspected to be resistant to ACCase-inhibiting herbicides since they survived repeated post-emergence clodinafop-propargyl application at the recommended dosage (64 g ai ha^−1^). Historical records showed that the fields had experienced previous applications of ACCase-inhibiting herbicides for more than 5 years. The populations were identified by the codes WR_1_, WR_2_, WR_3_, and WR_4_. Seeds of a susceptible population (S) were also collected from a wasteland in Khuzestan province where herbicide had never been used.

Because the populations were collected from different areas, the collected seeds were first cultivated under the same conditions in Pakdasht (35.4669° N, 51.6861 E), Tehran, Iran, in 2015 and 2017 (one collection each year) in order to eliminate the environmental effects on seed production. To relieve dormancy, the seeds were stratified at 5 °C for 4 weeks. Then, they were planted in 2 × 3 cm^2^ pots containing loam soil (30% sand, silt 35%, clay 35%) and decomposed manure; pH 7.5. The pots were irrigated to field capacity every 4 days. The plants were grown in a greenhouse with 25/18 °C of day/night temperature and natural photoperiod. To ensure that there was no chance of cross-pollination, spikes were covered with paper bags during the flowering stage. The produced seeds from each biotype were then used in whole plant dose response assay and first germination experiment conducted in 2015. To increase the accuracy of germination traits assessment, seeds were grown for another generation under the mentioned similar conditions. The germination characteristics of the seeds were re-evaluated in 2017.

### 2.2. Whole Plant Dose Response Assay

Eight seeds from each biotype were sown in 30 × 35 cm^2^ plastic pots filled with loam soil (30% sand, silt 35%, clay 35%) and decomposed manure; pH 7.5). The pots were arranged in a completely randomized design with four replications. Thinning was applied to reduce seedlings number to four. Clodinafop-propargyl treatments were applied at 3–4 leaf stages with 0, 1, 2, 4, 8 and 16 times of the recommended field dose (64 g ai ha^−1^). Four weeks after herbicide application, *A. ludoviciana* survival and aboveground biomass were recorded as a percentage of the untreated individuals. The four parameters log-logistic curve (Equation (1)) was fitted to the data using the R statistical software [34] with the add-on package *drc* [35].

(1) $$Y=\frac{D-C}{1+exp\left[b(loglog\;\left(X\right)-loglog\;({ED}_{50}))\right]}$$

where *Y* is the biomass reduction, *D* is the upper limit, *C* is the lower limit, *ED*_50_ is the dosage (g ai ha^−1^) that reduced fresh weight by 50%, b is the relative slope around *ED*_50_, and *X* is the herbicide dose (g ai ha^−1^). To describe the degree of resistance for biotypes, the ratio of absolute *ED*_50_ values of each resistant biotype to susceptible one was used to calculate the resistance index (RI) [36].

### 2.3. Investigating Molecular Basis of Resistance

The seeds (four seeds from each biotype) were sown in plastic pots (20 × 25 cm) filled with loam soil mixture, and the plants were maintained in a greenhouse at 18 and 22 °C in artificial light under a 16/8-h (day/light) photoperiod for leaf sampling. The greenhouse was located at Aburaihan Campus, University of Tehran (35°28′ N, 51°36′ E and 1020 masl), Iran. To assess the probability of mutation of the site of action being responsible for the resistance, genomic DNA was isolated from leaf tissues at 3-leaf to 4-leaf stage of three individual plants of each putative resistant and susceptible wild oat biotype using a modified cetyltrimethylammonium bromide (CTAB) extraction procedure [37]. The isolated DNA was quantified by measuring the absorbance at 260 nm by a spectrophotometer. Cleaved Amplified Polymorphic Sequences (CAPS) and derived Cleaved Amplified Polymorphic Sequences (dCAPS) molecular methods were used to identify the locations of four previously known mutations (Isoleucine-2041-Asparagine, Cysteine-2088-Arginine, Isoleucine-1781-Leucine, and Aspartic acid-2078-Glycine) responsible for target site-based herbicide resistance in the carboxyl transferase (CT) domain of the chloroplastic ACCase enzyme of the above-mentioned biotypes [4]. Primers and restriction enzymes used in CAPS and dCAPS methods are shown in Table 2 and Table 3, respectively.

### 2.4. Germination and Seedling Growth

To investigate the germination differences of the biotypes, two-seed bioassay was conducted under different water potential conditions. As mentioned in the seed source section, the seeds produced under the same conditions in 2015 and the next generation in 2017 were studied in two separate experiments (Hereinafter referred to as the first and second experiments, respectively). Seed water content percentage was determined in four samples (100 seeds per sample) of each biotype by weighing fresh seeds (w1) and oven-dried seeds (w2) as follows (Equation (2)):

(2) Seed water content=(w1−w2)/w1

Then, ISDW was measured before starting the experiment by weighing fresh seeds minus seed water weight. Three replicates of 25 seeds for each biotype were germinated in Petri dishes of 150 mm in diameter on filter paper (Whatman No. 1) at 20 °C and dark, at five water potentials (0, −0.15, −0.30, −0.45, and −0.6 MPa). Polyethylene glycol 6000 (PEG) was used to maintain water potentials determined according to Michel and Kaufmann [38]. Filter papers were soaked at the desired PEG solutions for 24 h, after which seeds were placed in Petri dishes and sealed. When the moisture of the Petri dishes decreased as affected by evaporation, seeds were moved into new Petri dishes and new solutions. Seed germination was assessed twice a day and the seeds with a radicle ≥2 mm long were considered germinated. Seeds were inspected for up to two weeks.

Heterotrophic seedling growth was evaluated after 14 days. The seedlings and seed remnants were separated first, and then weighed using an analytical balance with a milligram scale to determine the dry weight of seedlings (SLDW) and dry weight of seed remnants (FSDW). The weight of the mobilized seed reserve (MSR), the conversion efficiency of mobilized (CEM), and the fraction of mobilized seed reserve (FSR) were calculated as follows (Equations (3)–(5)) [4]:

(3) MSR=ISDW−FSDW

(4) CEM=SLDW/MSR

(5) FSR=MSR/ISDW

Data from the heterotrophic seedling growth test were analyzed as a combined analysis of multiple experiments (factors: experiment, biotype and water potential) and biotype × water potential interaction were compared by least significant difference (LSD) test if the interaction was significant by F test. Statistical analyses were performed with SAS software (Ver. 9.4.).

The hydrotime model was used to describe seed germination response to different water potentials (ψ; MPa) for each biotype [21,22]. The hydrotime constant (θ_H_; MPa-hours) was obtained as the following equation (Equation (6)):

θ_H_ = (Ψ − Ψ_b(g)_)tg (6)

where ψ_b(g)_ is the base water potential (MPa) for a specific germination percentage (g), and tg is the time (hours) to g percentage of germination for each biotype. Typically, variation in ψ_b_ follows a normal bell curve within a seed population [39]. Thus, the hydrotime model parameters were determined by repeated probit analysis using Equation (7), and the θ_H_ varied until the best fit was obtained for each biotype [20,23,39]:

probit (g) = [Ψ − (θ_H_/tg) − Ψ_b(50)_]/σ_Ψb_ (7)

where ψ_b(50)_ is the median, ψ_b_, and σ_ψb_ is the standard deviations in ψ_b_ among the seeds within the biotypes. The calculations were performed for each replication separately to estimate standard errors of the parameters. The Excel software was used for all calculations. 

## 3. Results and Discussion

### 3.1. Whole Plant Dose Response Assay

**Figure 1:**
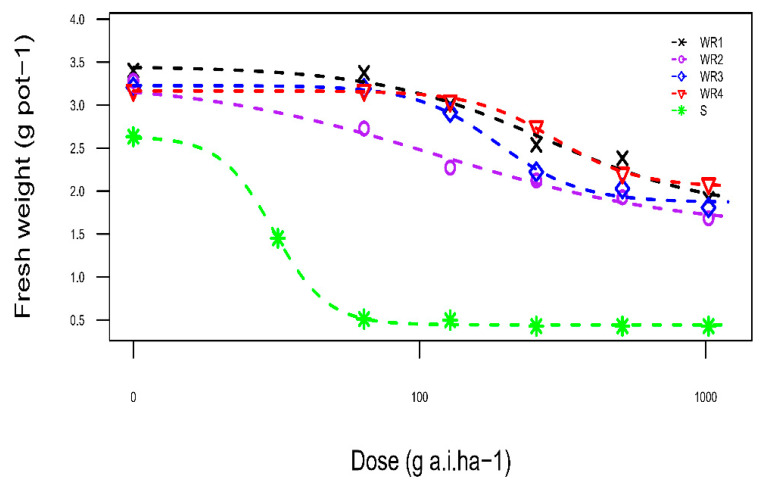
Effect of different concentrations of clodinafop- propargyl herbicide on aboveground fresh weight of susceptible and resistant biotypes. Symbols and lines represent actual and estimated response of resistant and susceptible biotypes, respectively. The symbols represent the mean of four replicates. The plants were grown in a greenhouse.

The resistant biotypes survived 4 weeks after all treatments while no susceptible biotype plant survived. The log-logistic model (Equation (1)) fitted adequately to the response of shoot fresh weight of biotypes to increasing rates of clodinafop-propargyl (Figure 1). The susceptible biotype was completely controlled at a rate lower than the recommended, suggesting that the S biotype is highly susceptible to clodinafop-propargyl rates (Table 4). The dose-results indicated that all identified resistant biotypes are classified as very highly resistant (RI > 100) to clodinafop-propargyl [40].

Resistance of *A. ludoviciana* to clodinafop-propargyl and other ACCase inhibitor herbicides had been reported in different countries in the world such as Australia, France [2] and Turkey [41]. The most common reason for weed resistance evolution is that herbicide application is the sole method of weed control combined with little or no variety in agronomic practices [42]. ACCase inhibitor herbicides have been extensively used by farmers for a decade as a practical selective herbicide to control weedy grasses in wheat production regions of Iran, especially in Khuzestan Province [43]. The results of screening studies confirmed the evolution of resistance in winter wild oat to ACCase inhibitors in Iran [32,44,45,46]. In a survey, Zand et al. [47] also characterized 52% of clodinafop-resistant *A. ludoviciana* populations in 50 farmer’s fields in Khuzestan Province.

### 3.2. Molecular Basis for Resistance

**Figure 2:**
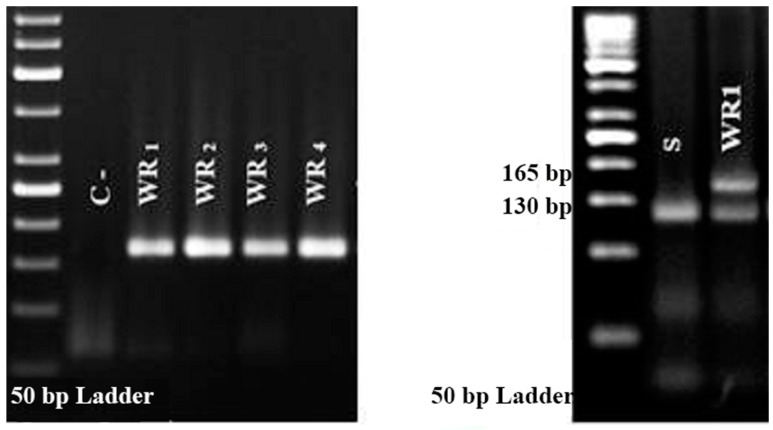
Polymerase chain reaction (PCR) (**Left**), and dCAPS analysis of individual *Avena sterilis* ssp. *ludoviciana* (**Right**). The size of the restriction enzyme (NsiI) digested fragment is 165 bp.

The CAPS markers and dCAPS markers were amplified in all biotypes, along with the desired region of the ACCase enzyme (Figure 2). Results of CAPS and dCAPS detected the substitution of Isoleucine for Leucine at position 1781 in the CT domain of the acetyl-CoA carboxylase gene in WR_1_ and WR_4_ resistant biotypes. However, this amino acid substitution was not confirmed in the other resistant biotypes, WR_2_, and WR_3_ (Figure 2). Results of enzyme restriction with NsiI also showed WR_1_ and WR_4_ biotypes were heterozygous for the resistant 1781- Leucine (Figure 2).

In most cases, resistance to ACCase inhibitors has been reported to be a result of target site mutations and insensitivity of ACCase [48,49]. It has been reported that I1781L substitution is the most frequent one conferring resistance to all three ACCase chemical herbicide families [12]. Yu et al. [4] found ACCase mutation in resistant *Lolium* populations. They detect 1781-Leu allele in many individuals (71%) of clethodim-resistant populations. These genotypes also exhibited cross resistance to aryloxyphenoxypropionate herbicides such as clodinafop, diclofop and fluazifop.

The resistance levels of biotypes containing I1781Le mutation were very high (RI > 214.22). Therefore, it was concluded that this substitution resulted in a high level of clodinafop-propargyl resistance in these populations. It was found that ACCase target site mutations conferred very high levels of resistance [50]. Resistance mechanism in other resistant biotypes (WR_2_ and WR_3_) that did not represent any point mutation in studied codons (Isoleucine-2041-Asparagine, Cysteine-2088-Arginine, Isoleucine-1781-Leucine, and Aspartic acid-2078-Glycine) was probably due to a mutation in other locations in the CT domain of the ACCase enzyme. Seven sites were reported to confer ACCase-inhibitor resistance in various weed species among the 13 conserved amino acid substitutions [6,51,52]. The results of a biochemical-based investigation of resistance to the acetyl-coenzyme A carboxylase (ACCase)-inhibiting herbicide diclofop-methyl in a resistant *Avena* population established that one or at least two independent resistance mechanisms (target-site ACCase resistance mutations and non–target-site enhanced rates of herbicide metabolism) can confer resistance in individual wild oat populations [53].

### 3.3. Germination and Seedling Growth 

**Figure 3:**
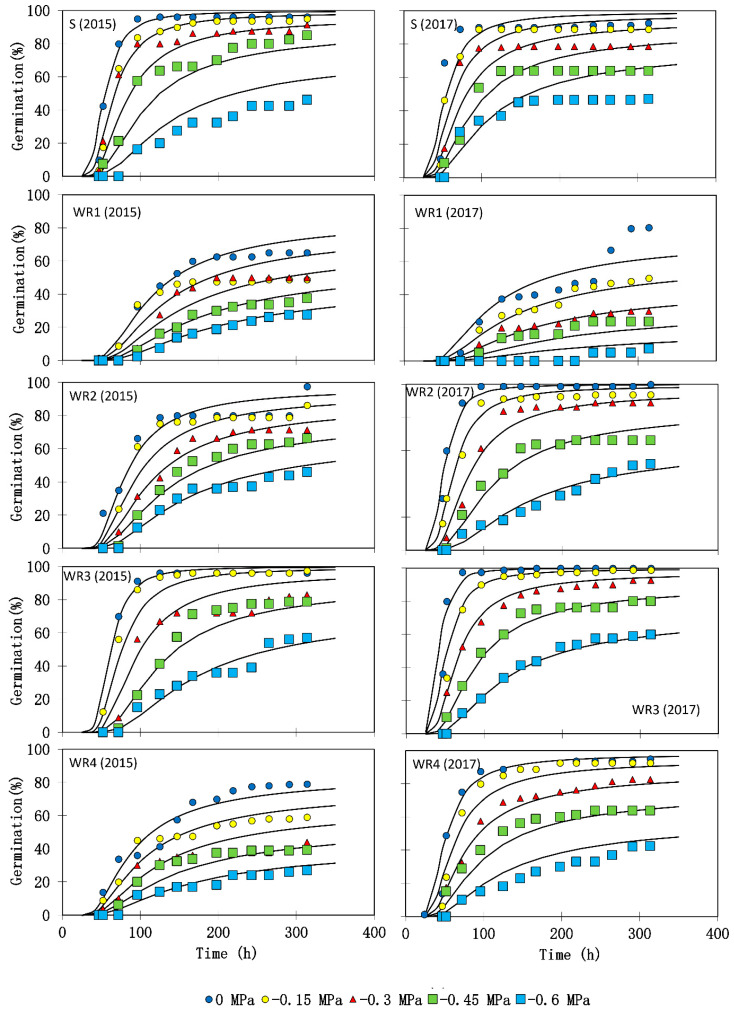
Germination time courses of five *Avena sterilis* ssp. *ludoviciana* biotypes (susceptible biotype (S) or resistant biotypes (WR_1_–WR_4_)). Symbols indicate interpolations of observed germination data and lines germination time courses predicted by the hydrotime model based on parameter estimates in Table 6. The symbols represent the mean of three replicates.

Results indicated that the total germination percentage differed among the biotypes (Figure 3). Water stress significantly decreased germination percentage (Figure 3) and seedling growth in all the biotypes (Table 5). The highest hydrotime constants (θ_H_) were observed in WR_1_ in both experiments (Table 6). The median base water potentials [ψ_b(50)_] of the two experiments were significantly higher (less negative) in WR_1_ and WR_4_ biotypes as compared with other resistant biotypes (Table 6). The lowest median base water potential (−0.79 MPa in the first experiment and −0.91 MPa in the second experiment) was observed in the susceptible biotype. The values of σ_ψb_ for each biotype are indicated in Table 6. 

**Figure 4:**
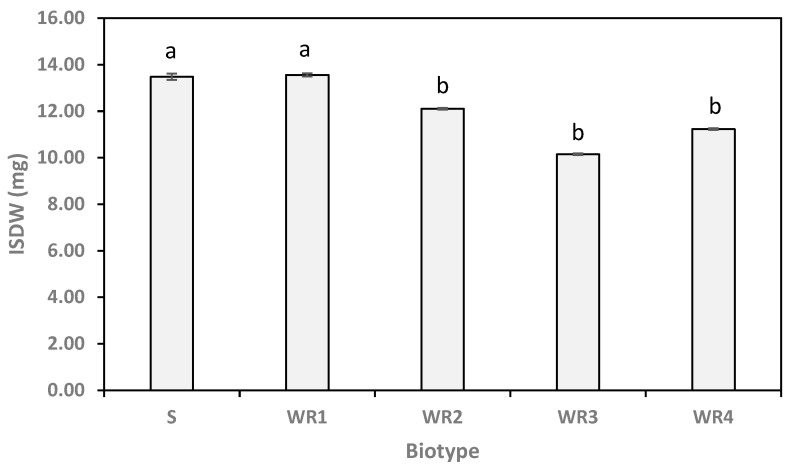
The average of initial seed dry weight (ISDW) among five *Avena sterilis* ssp. *ludoviciana* biotypes (susceptible biotype (S) or resistant biotypes (WR_1_–WR_4_)) produced in 2015 and 2017. The symbols represent the mean of three replicates. Error bars represent standard deviations. Means followed by the same letter are not significantly different according to the least significant difference (LSD) test.

As shown in Figure 4, ISDW significantly differed among biotypes, ranging from 13.56 mg (for WR_1_) to 10.15 mg (for WR_3_). Heterotrophic seedling growth test (except for ISDW) indicated significant interaction of the biotype and the water potential (Table 5). The FSDW ranged from 3.03 mg (for WR_3_ in 0 water potential) to 11.58 mg (for WR_1_ in −0.6 water potential) (Table 7). The SLDW changed significantly among biotypes in each water potential; and with the water potential decreasing, resistant biotypes, especially WR_1_, lost seedling growth. Results indicated that biotypes used MSR variously, and had significantly different CEM seed reserve to seedling tissue (Table 7). The CEM values ranged from 0.00 (for WR_1_) to 0.92 (for WR_3_) mg mg^−1^. In all water potential different resistant biotypes had the highest mobilized FSR and the S biotype the lowest value in 0 and −0.15 MPa (Table 7).

Resistant biotypes (in particular WR_1_ and WR_4_) had higher base water potential than the susceptible one, showing that resistant biotypes require more soil water for germination initiation. Hydrotime changes were different in the two experiments; in the first experiment, the hydrotime constant was higher in resistant biotypes than in the susceptible one, implying faster germination in the former, but in the second experiment, only WR_1_ had higher hydrotime than the susceptible biotype. Thus, it seems that the changes of hydrotime are not affected by herbicide resistance. Opposite results, as observed in the first experiment, were reported before, in which faster germination was found in resistant biotypes of *Kochia scoparia* in comparison with the susceptible biotype [16]. In addition, the results of seed biology investigation of sulfonylurea-resistant prickly lettuce (*Lactuca serriola*) and susceptible biotypes showed that germination rate of the resistant biotype was 100% faster than the susceptible one [15], whereas slower germination had been detected in two resistant species of *Amaranthus* [14] and *Phalaris minor* [17]. The resistance mechanism and level of clodinafop-propargyl resistance are believed to account for the vast majority of the variability between resistant biotypes of *A. ludoviciana*. Since the biotypes were collected in a province with the same climatic conditions, there could not be any other important factor causing these large changes in biotypes.

Although *A. ludoviciana* grows in drylands and can survive and produce seeds under water stress [54], there are several advantages of a higher base water potential in the resistant biotype of *A. ludoviciana* than in the susceptible one as follows: Seedling emergence of winter annual weeds such as *A. ludoviciana* is not limited by soil moisture [54], and resistant biotypes require more water to germinate, so seedlings are more likely to grow under more moist conditions. In this condition, crop irrigation has a key impact on seedling emergence of weeds. If the base water potential is very low (e.g., −1.5 MPa), it is possible to have seedling emergence under a low soil moisture condition and a further reduction in soil moisture in the following days will cause the emerging seedlings to die. Due to their higher base water potential, seeds of resistant *A. ludoviciana* biotypes have to wait for the first irrigation; thus, they emerge simultaneously with the crop sowing date and do not experience water stress. Indeed, this is an avoidance mechanism to cope with water stress. 

In our study, the susceptible biotype had a significantly higher grain weight than three resistant biotypes (WR_2_, WR_3_, and WR_4_) but did not differ significantly from WR_1_ (Figure 4). The grain weight of diclofop-methyl resistant individual plants of *Lolium rigidum* was significantly lower than that measured in susceptible plants. Early vigor of plants of resistant populations studied was also significantly lower than that measured in a susceptible population [55]. However, it was reported that there were no significant differences in one thousand seeds’ weight of resistant *L. rigidum* populations containing Ile1781Leu and Ile 2041Asn mutations when compared to a sensitive population [56].

Having a higher base water potential and lower seed weight in biotypes containing Il-1781-L mutation compared to non-mutant biotypes can be considered as one of the effects of this amino acid substitution. However, in the case of the hydrotime, since this trait in one of the resistant mutant-biotypes (WR_1_) is not higher than two non-mutant resistant biotypes, this attribute cannot be related to the mentioned mutation.

The SLDW significantly varied among biotypes; WR_1_ and WR_4_ had the lowest SLDW among them. As indicated before, heterotrophic seedling growth is influenced by two components, MSR and CEM. Results showed that MSR and CEM changed significantly among the biotypes. In this regard CEM was lower in WR_1_ and WR_4_ than in the others and two components of MSR were significantly different. ISDW was significantly lower in resistant biotypes (except for WR_1_) than in the S biotype, but mobilized FSR was significantly higher in resistant biotypes (except for WR_1_ under water stress conditions) than in susceptible biotypes (Table 7). This shows that resistant biotypes (especially at the 0 MPa) used more seed reserve to produce seedling growth and needed more energy.

Our results revealed that all the suspected resistant biotypes of *A. ludoviciana* studied in this research were very highly resistant to clodinafop-propargyl. Two resistant biotypes contained Isoleucine to Leucine amino acid substitution and no mutations were found in two other biotypes. The herbicide-resistant biotypes had a higher base water potential and higher hydrotime (in WR_1_ in both experiments and in all biotypes in the first experiment) for germination than the susceptible biotype. This shows that the latter can germinate, for a shorter time, at a lower soil water content than resistant biotypes. ACCase-resistant biotypes containing a mutation also used more seed reserve to start seedling growth. These results indicated that target site mutation at Ile1781 codon position could make ACCase-resistant biotypes less competitive than the S biotype; but they have become resistant to herbicides rather than growing faster.

Different methods can be used to control herbicide-resistant weeds, specifically ACCase-resistant *A. ludoviciana*. Crop management practices that lead to rapid stand establishment and canopy development minimize the effect of weeds. A number of management practices are necessary to control the growth of this weed, including crop rotation, planting certified seed, improving seedbed preparation, seeding at the correct rate, depth, and time of year. We believe that by designing and implementing appropriate management operations, such as stale seedbeds, at the right time, small differences between resistant and susceptible winter wild oats can be very useful in the control of resistant plants. A weed management program that includes monitoring weeds in the fields before and during the cultivation season is necessary to achieve success and does not use herbicides unless absolutely necessary. Using a herbicide over a long period of time increases herbicide resistance. To counteract this, it is recommended that herbicides be changed every few years.

## Figures and Tables

**Table 1:** Field Locations and History of Studied Wild Oat (*Avena sterilis* ssp. *ludoviciana*).

Table. *Cont.*	City	Location	The History of Grass Herbicide Application (Last 5 Years before Seed Sampling)
S	Andimeshk	32.45° N, 48.35° E	None
WR_1_	Andimeshk	32.45° N, 48.35° E	Clodinafop-propargyl
WR_2_	Shush	32.20° N, 48.25° E	Clodinafop-propargyl
WR_3_	Ahvaz	31.32° N, 48.67° E	Clodinafop-propargyl
WR_4_	Andimeshk	32.45° N, 48.35° E	Clodinafop-propargyl

**Table 2:** Sequences of CAPS and D CAPS Primers.

Primer	Sequence 5′-3′	Usage	Reference
ACCF1	CACAGACCATGATGCAGCTC	CAPS for 2041and 2088	[4]
ACCR1	CTCCCTGGAGTTGTGCTTTC	-	
NsiI1781f	CTGTCTGAAGAAGACTATGGCCG	dCAPS for 1781	[1]
NsiI1781r	AGAATACGCACTGGCAATAGCAGCACTTCCATGCA	-	

**Table 3:** Restriction Sites of CAPS and D CAPS Restriction Enzymes.

Enzyme	Commercial Isoschizomers	Restriction Site	Technique	Reference
NsiI	*Ava*III, *Eco*T22I, *Mp*h11031, *Zsp*2I	5′-ATGCA^T-3′ 3′-T^ACGTA-5′	dCAPS (1781)	[1]
EcoRI	*Fun*II	5′-G^AATTC-3′ 3′-CTTAA^G-5′	CAPS (2041)	[4]
EcoRV	*Eco*32I	5′-GAT^ATC-3′ 3′-CTA^TAG-5′	dCAPS (2078)	[4]
Eco47III	*Afe*I, *Aor*51HI, *Fun*I	5′-AGC^GCT-3′	CAPS (2088)	[4]

**Table 4:** Parameter estimates (SE) of four-parameter log-logistic model and resistance indices (RI) from whole plant bioassay of the suspected resistance (WR_1_, WR_2_, WR_3_, WR_4_) and susceptible (S) biotypes.

Biotype	B	C (g.pot^−1^)	D (g.pot^−1^)	Absolute ED_50_ (g a.i. ha^−1^)	RI
WR_1_	1.45 (0.58)	1.74 (0.42)	3.45 (0.11)	>1024 ^NA^	>214.22
WR_2_	1.00 (0.58)	1.55 (0.40)	3.28 (0.13)	>1024 ^NA^	>214.22
WR_3_	3.01 (1.25)	1.87 (0.12)	3.23 (0.11)	>1024 ^NA^	>214.22
WR_4_	4.67 (3.13)	2.04 (0.16)	3.16 (0.09)	>1024 ^NA^	>214.22
S	3.26 (2.93)	0.44 (0.06)	2.63 (0.13)	1.90	-

B: The relative slope around the parameter e, C is the lower limit, D is the upper limit, ED_50_ (absolute): Estimated by function *(ED (type="absolute"))* in the *drc* package of R software. RI = absolute ED_50_ Resistant population/absolute ED_50_ Susceptible population). ^NA^ Not possible to estimate the ED_50_ as plant fresh weight reduction was lower than 50% for all applied doses.

**Table 5:** Results of analysis of variance (mean squares) for initial seed dry weight (ISDW), seed remnants dry weight (FSDW), seedling dry weight (SLDW), the weight of mobilized seed reserve (MSR), the conversion efficiency of mobilized (CEM), and the fraction of seed reserve (FSR).

SOV	Df	ISDW	SLDW	FSDW	MSR	CEM	FSR
Experiment	1	0.0198	0.0035	0.220	0.373	0.0013	0.0019
Replication (Experiment)	2	0.0228	0.0909	0.335	0.387	0.0011	0.0024
Biotype	4	64.429 **	31.641 **	51.454 **	15.812 **	1.8749 **	0.0911 **
Water potential	4	0.0004	70.958 **	114.168 **	114.305 **	0.1843 **	0.8008 **
Bio × Exp	4	0.0004	0.0817	1.032	1.042	0.0155	0.0068
Bio × WP	16	0.0008	3.423 **	6.404 **	6.449 **	0.1286 **	0.0416 **
Exp × WP	4	0.0002	0.0551	0.898	0.893	0.0027	0.0052
Bio × Exp × WP	16	0.0005	0.0695	0.544	0.540	0.0071	0.0032
Residual	98	0.0074	0.2371	0.502	0.514	0.0114	0.0035

** Significant at 0.01 level of probability.

**Table 6:** Hydrotime constant (θ_H_), median base water potential (Ψ_b(50)_), standard deviation of the ψb(g) (σ_Ψb_). Coefficient of determination (*R*^2^) and Root Mean Square Error (RMSE)for susceptible (S) and resistant biotypes (WR_1_–WR_4_).

Biotype	θ_H_ (MPa-Hours)	Ψ_b(50)_ (MPa)	σ_Ψb_	RMSE	*R* ^2^
First experiment					
S	47.80 ± 6.34	−0.793 ± 0.012	0.268 ± 0.112	8.73	0.90
WR_1_	87.42 ± 5.87	−0.609 ± 0.024	0.529 ± 0.164	9.28	0.84
WR_2_	72.34 ± 4.45	−0.832 ± 0.008	0.431 ± 0.067	6.04	0.90
WR_3_	48.01 ± 5.12	−0.778 ± 0.009	0.239 ± 0.082	7.43	0.80
WR_4_	61.09 ± 6.78	−0.530 ± 0.010	0.502 ± 0.105	5.65	0.92
Second experiment					
S	48.99 ± 4.31	−0.908 ± 0.014	0.369 ± 0.089	10.56	0.81
WR_1_	51.41 ± 3.44	−0.280 ± 0.018	0.393 ± 0.077	5.63	0.82
WR_2_	34.41 ± 2.13	−0.701 ± 0.004	0.220 ± 0.084	4.71	0.95
WR_3_	31.15 ± 2.87	−0.750 ± 0.008	0.222 ± 0.094	5.27	0.95
WR_4_	36.31 ± 3.83	−0.687 ± 0.009	0.321 ± 0.075	6.05	0.93

**Table 7:** Initial seedling dry weight (SLDW), seed remnants dry weight (FSDW), the weight of mobilized seed reserve (MSR), the conversion efficiency of mobilized (CEM), and the fraction of seed reserve (FSR) for susceptible biotype (S) or resistant biotypes (WR_1_–WR_4_).

Water Potential (MPa)	Biotype	SLDW (mg)	FSDW (mg)	MSR (mg)	CEM (mg mg^−1^)	FSR (mg mg^−1^)
−0.6	S	2.12 ^a^	10.26 ^b^	3.22 ^a^	0.66 ^b^	0.24 ^a^
	WR_1_	0.00 ^c^	11.58 ^a^	1.98 ^bc^	0.00 ^c^	0.15 ^b^
	WR_2_	2.00 ^a^	8.82 ^c^	3.27 ^a^	0.53 ^b^	0.27 ^a^
	WR_3_	1.22 ^b^	8.75 ^c^	1.40 ^c^	0.89 ^a^	0.14 ^b^
	WR_4_	1.53 ^b^	8.76 ^c^	2.47 ^b^	0.63 ^b^	0.22 ^a^
−0.45	S	3.16 ^a^	9.45 ^b^	4.03 ^a^	0.79 ^a^	0.30 ^b^
	WR_1_	0.00 ^c^	10.80 ^a^	2.76 ^b^	0.00 ^c^	0.20 ^c^
	WR_2_	2.66 ^a^	7.31 ^d^	4.80 ^a^	0.59 ^b^	0.39 ^a^
	WR_3_	1.67 ^b^	8.26 ^c^	1.88 ^c^	0.92 ^a^	0.19 ^c^
	WR_4_	1.87 ^b^	8.14 ^c^	3.10 ^b^	0.61 ^b^	0.28 ^b^
−0.3	S	3.41 ^b^	8.83 ^b^	4.65 ^b^	0.74 ^ab^	0.35 ^bc^
	WR_1_	0.00 ^d^	11.10 ^a^	2.44 ^d^	0.00 ^c^	0.18 ^d^
	WR_2_	4.66 ^a^	6.63 ^d^	5.49 ^a^	0.86 ^a^	0.45 ^a^
	WR_3_	2.90 ^b^	6.17 ^d^	3.96 ^bc^	0.74 ^ab^	0.39 ^ab^
	WR_4_	2.04 ^c^	7.95 ^c^	3.29 ^c^	0.64 ^b^	0.29 ^c^
−0.15	S	3.97 ^b^	8.51 ^a^	4.98 ^b^	0.80 ^a^	0.37 ^c^
	WR_1_	3.27 ^c^	7.31 ^b^	6.26 ^a^	0.52 ^c^	0.46 ^b^
	WR_2_	5.37 ^a^	5.68 ^c^	6.42 ^a^	0.84 ^a^	0.53 ^a^
	WR_3_	3.93 ^b^	5.50 ^c^	4.67 ^b^	0.84 ^a^	0.46 ^b^
	WR_4_	2.87 ^c^	6.85 ^b^	4.37 ^b^	0.67 ^b^	0.39 ^c^
0	S	4.80 ^b^	8.11 ^a^	5.36 ^d^	0.89 ^a^	0.40 ^c^
	WR_1_	5.03 ^b^	3.84 ^bc^	9.72 ^a^	0.53 ^c^	0.72 ^a^
	WR_2_	7.10 ^a^	3.78 ^bc^	8.33 ^b^	0.85 ^a^	0.69 ^a^
	WR_3_	5.04 ^b^	3.03 ^c^	7.12 ^c^	0.71 ^b^	0.70 ^a^
	WR_4_	3.79 ^c^	4.50 ^b^	6.74 ^c^	0.56 ^c^	0.59 ^b^

Same letters within the same column for each water potential indicate no significant difference at *p* = 0.05.

## References

[1] Kaundun S.S., Windass J.D. (2006). Derived cleaved amplified polymorphic, a simple method to detect a key point mutation conferring acethyl CoA carboxylase inhibitor herbicide resistance in grass weeds. Weed Res..

[2] Heap I. The International Survey of Herbicide Resistant Weeds. http://www.weedscience.com.

[3] Lonhienne T., Garcia M.D., Pierens G., Mobli M., Nouwens A., Guddat L.W. (2018). Structural insights into the mechanism of inhibition of AHAS by herbicides. Proc. Natl. Acad. Sci. USA.

[4] Yu Q., Collavo A., Zheng M.Q., Owen M., Sattin M., Powles S.B. (2007). Diversity of acetyl-coenzyme A carboxylase mutations in resistant *Lolium* populations: Evaluation using clethodim. Plant Physiol..

[5] Delye C., Michel S., Berard A., Chauvel B., Brunel D., Guillemin J.P., Le Corre V. (2010). Geographical variation in resistance to acetyl-coenzyme A carboxylase-inhibiting herbicides across the range of the arable weed *Alopecurus myosuroides* (blackgrass). New Phytol..

[6] Powles S.B., Yu Q. (2010). Evolution in action: Plants resistant to herbicides. Ann. Rev. Plant. Biol..

[7] Coustau C., Chevillon C., French-Constant R. (2000). Resistance to xenobiotics and parasites: Can we count the cost?. Trends Ecol. Evol..

[8] Pavlicev M., Wagner G.P. (2012). A model of developmental evolution: Selection, pleiotropy and compensation. Trends Ecol. Evolution..

[9] Jang S., Marjanovic J., Gornicki P. (2013). Resistance to herbicides caused by single amino acid mutations in acetyl-C o A carboxylase in resistant populations of grassy weeds. New Phytol..

[10] Benakashani F., Zand E., Naghavi M.R., Sasanfar H. (2015). Mutations in Acetyl-CoA Carboxylase enzyme, mechanism of cross-resistance in wild oat (*Avena ludoviciana* Deuri.) biotypes to ACCase inhibitor herbicides. Iran J. Weed Sci..

[11] Sasaki Y., Nagano Y. (2004). Plant acetyl-CoA carboxylase: Structure, biosynthesis, regulation, and gene manipulation for plant breeding. Biosci. Biotechnol. Biochem..

[12] Kaundun S.S. (2014). Resistance to acetyl-CoA carboxylase-inhibiting herbicides. Pest Manag. Sci..

[13] Park K.W., Mallory-Smith C.A. (2005). Multiple herbicide resistant in downy brome (*Bromus tectorum*) and its impact on fitness. Weed Sci..

[14] Weaver E.S., Thomas G. (1986). Germination responses to temperature of atrazine-resistant and -S biotype of two pigweed (*Amaranthus*) species. Weed Sci..

[15] Alcocer-Ruthling M., Thill D.C., Shafii B. (1992). Seed biology of sulfonylurea- resistant and -S biotype of prickly lettuce (*Lactuca serriola*). Weed Technol..

[16] Dyer W.E., Chee P.W., Fay P.K. (1993). Rapid germination of sulfonylurea-resistant *Kochia scoparia* L. accessions is associated with elevated seed levels of branched chain amino acids. Weed Sci..

[17] Torres-Garcia J.S., Uscanga-Mortera E., Trejo C., Conde-Martinez V., Kohashi-Shibata J., Núñez-Farfán J., Martínez-Moreno D. (2015). Effect of herbicide resistance on seed physiology of *Phalaris minor* (*Little seed canarygrass*). Bot. Sci..

[18] Osipitan O.A., Dille J.A. (2017). Fitness outcomes related to glyphosate resistance in kochia (*Kochia scoparia*): What life history stage to examine?. Front. Plant Sci..

[19] Batlla D., Bench-Arnold R.L. (2004). A predictive model for dormancy loss in *Polygonum aviculare* L. seeds based on changes in population hydrotime parameters. Seed Sci. Res..

[20] Soltani E., Gruber S., Oveisi M., Salehi N., Alahdadi I., Javid M.G. (2017). Water stress, temperature regimes, and light control secondary dormancy induction and loss in *Brassica napus* L. seeds. Seed Sci. Res..

[21] Gummerson R.J. (1986). The effect of constant temperatures and osmotic potentials on the germination of sugar beet. J. Exp. Bot..

[22] Bradford K.J. (2002). Applications of hydrothermal time to quantifying and modeling seed germination and dormancy. Weed Sci..

[23] Soltani E., Farzaneh S. (2014). Hydrotime analysis for determination of seed vigor in cotton. Seed Sci. Technol..

[24] Soltani A., Gholipoor M., Zeinali E. (2006). Seed reserve utilization and seedling growth of wheat as affected by drought and salinity. Environ. Exp. Bot..

[25] Cheng X., Cheng J., Huang X., Lai Y., Wang L., Du W., Zhang H. (2013). Dynamic quantitative trait loci analysis of seed reserve utilization during three germination stages in rice. PLoS ONE.

[26] Seyyedi S.A., Khajeh-Hosseini M., Moghaddam P.R., Shahandeh H. (2015). Effects of phosphorus and seed priming on seed vigor, fatty acids composition and heterotrophic seedling growth of black seed (*Nigella sativa* L.) grown in a calcareous soil. Ind. Crop. Prod..

[27] Zheng Y.L., Ma H.C. (2014). Effects of seed aging on seed germination and seed reserve utilization in mumian. HortTechnology.

[28] Castellanos-Frías E., García de León D., Pujadas-Salva A., Dorado J., Gonzalez-Andujar J.L. (2014). Potential distribution of *Avena sterilis* L. in Europe under climate change. Ann. Appl. Biol..

[29] Beckie H.J., Warwick S.I., Sauder C.A. (2012). Basis for herbicide resistance in Canadian populations of wild oat (*Avena fatua*). Weed Sci..

[30] Torner C., Gonzalez-Andujar J.L., Fernandez-Quintanilla C. (1991). Wild oat (*Avena sterilis* L.) competition with winter barley: Plant density effects. Weed Res..

[31] Vidal R.A., Fleck N.G. (1997). The risk of finding herbicide resistant weed biotypes. Planta Daninha.

[32] Zand E., Benakashani F., Alizadeh H.M., Soufizadeh S., Ramezani K., Maknali S., Fereydounpoor M. (2006). Resistance to aryloxyphenoxypropionate herbicides in wild oat (*Avena ludoviciana*). Iran J. Weed Sci..

[33] Gherekhloo J., Oveisi M., Zand E., Deprado R. (2016). A review of herbicide resistance in Iran. Weed Sci..

[34] R Core Team (2013). R: A Language and Environment for Statistical Computing.

[35] Ritz C., Streibig J.C. (2005). Bioassay analysis using R. J. Stat. Softw..

[36] Keshtkar E., Kudsk P., Mesgaran M.B. (2021). Perspective: Common errors in dose–response analysis and how to avoid them. Pest Manag. Sci..

[37] Christoffers M.J., Berg M.L., Messersmith C.G. (2002). An isoleucine to leucine mutation in acetyl-CoA carboxylase confers herbicide resistance in wild oat. Genome.

[38] Michel B.E., Kaufmann M.R. (1973). The osmotic potential of polyethylene glycol 6000. Plant Physiol..

[39] Dahal P., Bradford K.J. (1990). Effects of priming and endosperm integrity on seed germination rates of tomato genotypes: II Germination at reduced water potential. J. Exp. Bot..

[40] Beckie H.J., Tardif F.J. (2012). Herbicide cross resistance in weeds. Crop. Prot..

[41] Torun H., Uygur F.N. (2018). Determination and mapping of resistant wild oat (*Avena sterilis* L.) populations to most commonly used herbicides in wheat fields for Osmaniye, Turkey. Int. J. Agric. Sci..

[42] Beckie H.J., Hall L.M., Meers S., Laslo J.J., Stevenson F.C. (2004). Management practices influencing herbicide resistance in wild oat. Weed Technol..

[43] Deihimfard R., Zand E. (2005). Evaluating environmental impacts of herbicides on wheat agroecosystems in the provinces of Iran using EIQ model. Environ. Sci..

[44] Benakashani F., Zand E., Alizadeh H.M. (2007). Resistance of wild oat (*Avena ludoviciana*) biotypes to clodinafop-propargil herbicide. Appl. Entomol. Phytopathol..

[45] Benakashani F., Rahimian Mashhadi H., Zand E., Alizadeh H., Naghavi M.R. (2010). Investigation of the cross resistance to ACCase inhibitor herbicides in wild oat (*Avena ludoviciana* Durieu.) populations from Khuzestan province and chemical control of resistant populations. Iran J. Weed Sci..

[46] Rastgou M., Rashed M.H., Zand E., Nasiri M. (2006). Resistance of winter wild oat (*Avena ludoviciana* Durieu.) to aryloxyphenoxy propionate herbicides in wheat fields of Khuzestan province: First screening test. Iran J. Weed Sci..

[47] Zand E., Benakashani F., Baghestani M.A., Maknali A., Minbashi M., Soufizadeh S. (2007). Investigating the distribution of resistant wild oat (*Avena ludoviciana*) populations to clodinafop-propargil herbicide in South Western. Iran. Environ. Sci..

[48] Delye C. (2005). Weed resistance to acetyl coenzyme A carboxylase inhibitors: An update. Weed Sci..

[49] Kukorelli G., Reisinger P., Pinke G. (2013). ACCase inhibitor herbicides—selectivity, weed resistance and fitness cost: A review. Int. J. Pest Manag..

[50] Gaines T.A., Duke S.O., Morran S., Rigon C.A.G., Tranel P.J., Küpper A., Dayan F.E. (2020). Mechanisms of evolved herbicide resistance. J. Biol. Chem..

[51] Zhang X.Q., Powles S.B. (2006). The molecular bases for resistance to acethyl co-enzyme A carboxylase (ACCase) inhibiting herbicides in two target based resistant biotypes of annual ryegrass (*Lolium rigidum*). Planta.

[52] Delye C., Pernin F., Michel S. (2011). ‘Universal’ PCR assays detecting mutations in acetyl-coenzyme A carboxylase or acetolactate synthase that endow herbicide resistance in grass weeds. Weed Res..

[53] Ahmad-Hamdani M.S., Yu Q., Han H., Cawthray G.R., Wang S.F., Powles S.B. (2013). Herbicide resistance endowed by enhanced rates of herbicide metabolism in wild oat (*Avena* spp.). Weed Sci..

[54] Mahajan G., Loura D., Raymont K., Chauhan B.S. (2020). Influence of soil moisture levels on the growth and reproductive behavior of *Avena fatua* and *Avena ludoviciana*. PLoS ONE.

[55] Matzrafi M., Gerson O., Rubin B., Peleg Z. (2017). Different Mutations Endowing Resistance to Acetyl-CoA Carboxylase Inhibitors Results in Changes in Ecological Fitness of *Lolium rigidum* Populations. Front. Plant Sci..

[56] Sabet Zangeneh H., Mohammaddust Chamanabad H.R., Zand E., Asghari A., Alamisaeid K., Travlos I.S., Alebrahim M.T. (2016). Study of fitness cost in three rigid ryegrass populations susceptible and resistant to acetyl-CoA carboxylase inhibiting herbicides. Front. Ecol. Evol..

